# Local knowledge, pattern and diversity of use of *Sclerocarya birrea*

**DOI:** 10.1186/1746-4269-7-8

**Published:** 2011-02-01

**Authors:** Gerard N Gouwakinnou, Anne Mette Lykke, Achille E Assogbadjo, Brice Sinsin

**Affiliations:** 1 Laboratory of Applied Ecology, Faculty of Agronomic Sciences, University of Abomey-Calavi, Benin. 01 PO Box 526 Cotonou, Benin; 2 Department of Terrestrial Ecology, National Environmental Research Institute, Aarhus University, Vejlsøvej, 25, Dk-8600 Silkeborg, Denmark

## Abstract

**Background:**

Growing interest is on food tree species in general, and particularly indigenous fruit tree species in developing countries since they are inherent to most tropical landscapes and serve the dual function of local livelihood support and biodiversity conservation. It is therefore relevant to assess the level of integration of these species in local cultures and the factors affecting them. This study aims at assessing knowledge and uses of *Sclerocarya birrea *subsp. *birrea *and factors affecting the use values within and between communities.

**Methods:**

This study combines quantitative and qualitative ethnobotanical approaches to investigate uses and factors affecting the use value of *S. birrea *subsp. *birrea*. Nine group discussions as well as 161 individual interviews were held in the dry and typical Sudanian zones. Seven different ethnic groups were involved and the survey focused on local uses and perception of factors affecting the dynamics of *S. birrea*.

**Results:**

The species has a multitude of uses; all organs are used for more than 20 different purposes. The study highlights how gender, local availability, ethnicity and community location interact to influence the utilization value of the species. People living in drier areas with high occurrence of the *S. birrea *use it more than those living in wetter areas with low occurrence. While domestic and subsistence uses do not appear to threaten the species, carving, clearing and drought stand out as the major causes of its decline.

**Conclusions:**

Many factors and their interactions influence the use pattern of the species within and between communities. When compared to the level of exploitation of *S. birrea *subsp. *caffra *in southern Africa, the subspecies *birrea *is at this point relatively underutilized. A high commercial potential exists due to its simple propagation ability and makes it an interesting agroforestry resource.

## Background

Growing interest is on food tree species in general, and particularly indigenous fruit tree species in developing countries since they are inherent to most tropical landscapes and serve the dual function of local livelihood support and biodiversity conservation [1]. More information on these trees would enhance their value in agricultural landscapes by helping farmers improve their livelihoods and ensuring environmental sustainability. Therefore, there is a need to settle a general framework for the conservation of these tree species.

Understanding how a community uses a resource and what influences the level of its use is crucial for developing a framework for its sustainable use based on local demands [2]. Previous studies have revealed that both consumptive and non-consumptive values derived from plant species are influenced by many factors which can be of socio-cultural, economical or ecological importance [3-5]. These studies have come to the conclusion that factors such as gender, age, localization, ethnic affiliation, marketability and proximity with other ethnic groups can interact to influence the use value of a given plant species. Based on the hypothesis of "local apparency" [6] which considers plants as resource and herbivores as consumers, it was found that local availability of a plant can influence local consumers behavior [6,7]. However, relationship between local availability and use values was found to be weak in others cases [2,8]. Taking into account results from these previous studies which highlight that plant species are not valued equally by local communities, we have assumed that the patterns of use of *Sclerocarya birrea *would be influenced by the above-mentioned factors.

*Sclerocarya birrea *(A. Rich) Hochst is a species with multifaceted uses which is recognized as a commercially, medicinally and culturally important plant species in Africa [9,10]. It has been identified as one of the five fruit tree species that should be integrated in the domestication process in farming systems in Africa to support nutritional, health and income security [11]. However, while there is a real initiative to exploit the subspecies *caffra *in the eastern and southern Africa, the subspecies *birrea *remains very much underutilized and less studied in western Africa. Previous researches have documented the population structure and abundance of the species in relation to land use and variation in chemical and phenotypic characteristics of its fruits in West Africa [12-14], but less is known on the pattern of use of *S. birrea *and how its use value varies among various ethnic groups throughout its distribution range in West Africa. We aim at combining quantitative and qualitative ethnobotanical approaches to assess knowledge and uses of *Sclerocarya birrea *subsp. *birrea*. Specifically, we aim at assessing i) the various uses and knowledge on the species ii) how local people perceive the decline of the species and iii) the factors affecting the use values within and between communities.

## Methods

### Study species

*Sclerocarya birrea *(A.Rich) Hochst belongs to the Anacardiaceae family. The genus *Sclerocarya *is a strictly African/Malagasy. *S. birrea *is a small to medium-sized, usually dioecious tree. It can reach up to 20 m in height and 1.2 m in diameter. The bole is usually short with ramifications from two to four metres height when isolated [14,15]. Three subspecies of *S. birrea *are recognized throughout its distribution range [16]. This study focuses on *S. birrea *subsp. *birrea*, the western African taxon hereafter referred to as *S. birrea*.

### Study area

**Figure 1:**
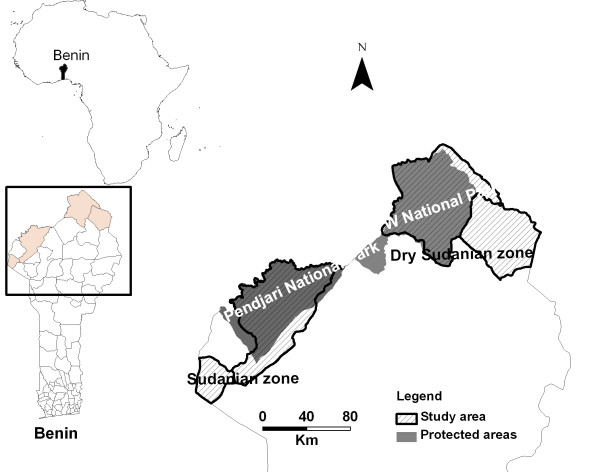
**Study sites with location of the two main protected areas**.

This study was carried out in the northern part of the Republic of Benin, between 10°17-12°25N and 0°45-3°51 E, which represents the distribution range of the species in Benin [17]. The study site stretches from the dry Sudanian climate in the northern side to the typical Sudanian climate in the southern side. The climate in southern side has about seven-month dry period with a mean annual rainfall of 1,000 mm. The mean annual temperature is 28°C and the vegetation is composed mostly of open shrub and tree savannas. The northern side has a dry sudanian climate with seven to eight month dry period and a mean annual rainfall of 650 mm. The mean annual temperature is about 30°C and the vegetation is characterized by grasslands and open shrub lands with sparse trees. We will hereafter refer to northern and southern zones as the dry Sudanian and Sudanian zones. The population of dry Sudanian zone was composed of the following ethnic groups; Dendi (Zerma), Fulani, Gourmanche and the Sudanian zone contained the Wama, Berba, M'bermè and Gourmanche ethnic groups. Fulani were pastoralists, while other ethnic groups were mostly agriculturalists. The study area encompassed the two main wildlife reserves in Benin: Pendjari National Park and W National Park (Figure 1).

### Data collection

**Figure 2:**
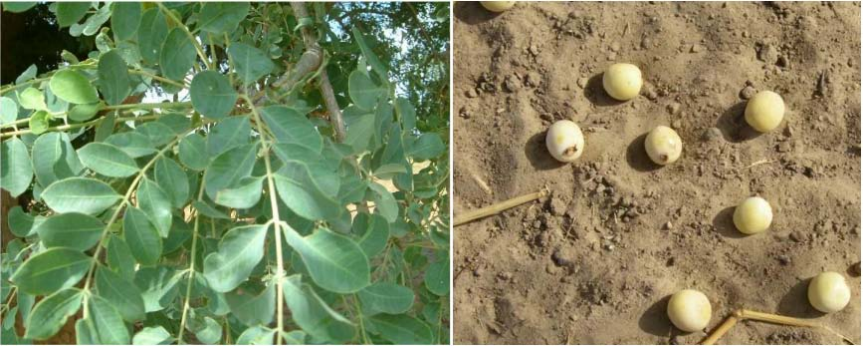
**Pictures of leaves and fruits of *Sclerocarya birrea *used for quick recognition of the species by local people during the survey**.

In the dry Sudanian zone, structured interviews were carried out with informants from representative socio-cultural groups: Dendi, Gourmanche, and Fulani. The sample included the major professional groups (farmers, traditional healers, cattle breeders, fishermen, and craftsmen) with respect to gender. Interviews were conducted either individually or in groups. Nine group discussions with a total of 107 participants as well as 161 individual interviews were conducted (Table 1). Participants in the group discussions were not chosen for individual interviews and vice versa. Some immigrant carvers from Niger (neighboring country of Benin) known to specifically seek *S. birrea *wood for carving in the dry seasons were included in the individual interviews. The survey focused on the investigation of local name of the species, which parts are used, the processing methods, the purpose of use, and how people perceived the decline of the species in time. To get an estimate of the presence index for the species in the survey area (mainly in farmland), each informant was asked whether he/she had at least one individual *S. birrea *tree on their farms and was asked to indicate the part of the plant that was used most frequently. In the Sudanian zone, the minimum sample size was calculated using exploratory analysis of data collected in the dry Sudanian zone. Pictures of the leaves and fruits of the species (Figure 2) were kept and shown to each informant as the species is less common in the Sudanian zone compared to the dry Sudanian zone. The objective was to dispel any confusion with other species. Interviews were conducted on an individual basis and were identical to those carried out in the first zone. Interviews were conducted in the local language (Table 1) through local translators.

**Table 1:** Different ethnic groups, local names of *Sclerocarya birrea *and details of sampling (individual and group interviews) in the study zones

Study zone	Sampled district	Ethnic group	Local name	Male	Female
Dry Sudanian	Mallanville	Dendi	Luley, Moru-Moru, Diney	30	5
	
		Dendi	Diney, Luley	83	32
		
		Gourmanche	Bunamagbu	17	4
	Karimama		Bunamangshiabu (for female)		
			Bunamangjabu (for male)		
		
		Fulani	Eedy	16	3

Sudanian	Cobly	M'Bermè	Ubamingbu	18	15
	
	Tanguiéta	Wama	Damahabu	10	2
		
		Berba	Namuak	15	4
		
		Gourmanche	Bunamangshiabu	12	2
			Bunamandjabu		

The sampling details presented for the dry sudanian zone include participants to group discussions held in this zone.

### Data analysis

We used a multiple use curve [18] to check whether the essential part of the available information on the species had been collected. This curve plotted the cumulative number of new uses recorded against the number of informants. The value at which the curve became asymptotic was taken as the minimal sample size for data collection in the second phase of the study. Multiple use curves were also derived from the second phase data. The multiple-use curve reached its asymptotic value at 33 interviews in the dry Sudanian zone and 17 interviews in the Sudanian zone.

To analyze the use values of the species, we regrouped the uses into broad categories, where each category contained uses of a similar nature. In this way, four main categories were created, namely; food, medicine, carving and firewood. Food and medicinal categories refer to use by both humans and animals.

Recorded uses were also divided into three separate temporal groups: past, current, and potential. Past uses were defined as those that had been used in the past but are no longer in use. Potential uses were defined as those that had a potential for future use and current uses were defined as those known to still be in use.

The use importance of the species per zone and comparison among sites were assessed using the use value (UV) [6] defined as:

$$\text{UV}=\frac{\sum \text{UVi}}{\text{N}}$$

UVi represents the use value of the species for a single informant. UVi is obtained as the sum of the number of different uses mentioned by the informant i, where N the total number of informants. Only current uses were included in the calculation of the use values to reflect the realized value. In order to satisfy statistical independence requirement, participants interviewed during group discussions were not included. Use values were calculated for each category of use and for all categories pooled together. Use values were compared between study zones by means of non-parametric Mann-Whitney U tests. The overall gender specific use value was also computed and compared using the student t-test.

The answer rates per specific use defined as the fidelity level (FL) [19] in each study zone have been expressed as:

$$\text{FL (\%)}=\frac{n}{\text{N}}*100$$

Where n is number of informants related to a specific use and N = total number of informants. We used the Fisher exact test (PROC FREQ in SAS) to test whether fidelity levels differed between study zones. The index of presence was taken as the percentage of informants having *S. birrea *on their farm.

## Results

### Ethnoecological knowledge on *Sclerocarya birrea*

#### Local names

Different local names were attributed to the species according to the ethnic groups (Table 1). The local name "Morou-Morou" in Dendi (Zerma) means sour and is descriptive of the taste of the fruits as perceived by local people. The name "Luley" was associated more with the kernel than to the entire fruit. In Gourmantché, the local names showed a distinction in the sex of the species: "Bunamangjabu" for male individuals and "Bunamanshiabu" for females.

#### Local perception of occurrence habitat and decline

*S. birrea *was reported to be present in open farmlands and in natural vegetation. When used for fence purposes, the species could regenerate in homesteads. Saplings were mainly reported to occur in fields left fallow. According to farmers, the species was scarce in quasi-permanently flooded areas, hills and gallery forest. The presence index of the species in agricultural farmlands was 74% in the dry Sudanian zone and 30% in the Sudanian zone.

**Figure 3:**
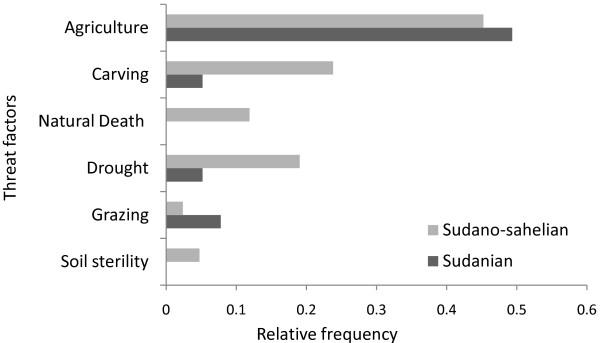
**Factors responsible of the decline in the population of *Sclerocarya birrea *according to local users**.

Almost all (98%) informants stated that the population of *S. birrea *has declined in recent times both in abundance and in distribution. The factors purportedly responsible for this decline were both anthropogenic (agriculture, felling for carving and grazing) and natural (decrease in soil fertility, natural death and drought) (Figure 3).

Agricultural factors contributing towards the decline included the destruction of natural habitat for conversion into cultivated land and by burning, ring-barking and removing *S. birrea *seedlings and saplings during farming activities.

### Diversity and pattern of use

#### Past, current and potential uses

**Figure 4:**
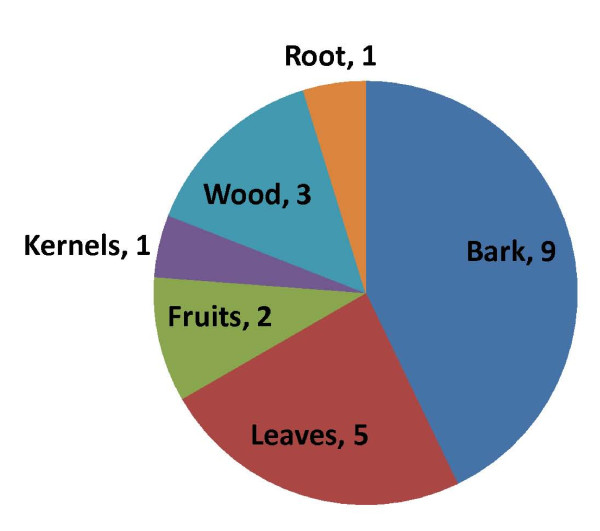
**Number of significant uses made of each organ of *Sclerocarya birrea***.

Although about 36 specific uses were recorded for the species throughout its distribution range, 21 of these were found to be significant (FL > 5%). The number of significant uses was greater in dry Sudanian zone (20 uses) than in the Sudanian zone (14 uses). The four defined use categories (food, medicine, firewood and carving) were represented in both studies zones. *S. birrea *was a multipurpose species with almost all organs used. The proportion of use of each plant organ varied per study zone. The bark, the wood and the leaves had multiple forms of use but the fidelity levels were rarely above 50%. The bark was the most frequently used organ, being used to treat ailments which are mostly bacteria-related (stomach aches, diarrhea, wounds, coughs) (Table 2). The fruits and kernels, on the contrary, were used almost exclusively for a single purpose and showed the highest fidelity level (up to 100%). The processing form, the use form, and the specific purpose of uses were fairly similar across study zones but there was a significant difference in the fidelity level of uses across study zones (Figure 4; Table 2).

**Table 2:** Different plant organs used, processing methods, forms of uses, purpose of use and fidelity level (FL) of uses of *Sclerocarya birrea *per study zone

Organ	Use category	Processing method	Form of use	Purpose of use	Fidelity Level (%)	
					
					Dry Sudanian zone (n = 84)	Sudanian zone (n = 77
				Malaria	46.43	3.90
				
				Stomach-ache	23.81*	35.06*
				
		Soak in cold water	Drink the liquid	Diarrhoea	11.90*	14.29*
				
Bark	Medicine	or		Haemorrhoids	5.95*	10.39
				
		Boil in water as infusion		Cough and tuberculosis	5.95*	10.39*
				
				Diabetes	5.91	-
			
			Rinse the mouth	Tooth ache	10.9	-
			
			Take a bath	Fortify infants	11.42	-
		
		Dry and reduce in powder	Sprinkle wounds	Wound healing	10.71	38.96

	Medicine	Boil in water as infusion or pound fresh	Wash the injured person or put pounded leaves in wounds	Human and animal wound healing	14.29	11.69
		
Leaves		Harvest young leaves and recuperate the sap	Put the sap on eyes	Sore eyes	28.57	-
	
	Food	Boil young leaves and mix with seasoning (dried peanut extract, red pepper, salt and other)	Eat in form of "leaf bundle"	Human nutrition	55.95	-
		
		Pound leaves with millet and transform in porridge	Drink the porridge	Milk production stimulation for nursing women	30.95 (100 for women)	1.30
	
	Pastoral	Harvest the leaves from trees	Give fresh leaves for cattle as forage	Cattle care	79.76	46.75

Fruits	Food	Remove the flesh or make a hole on fruit and extract juice	Eat Drink juice	Human and animal nutrition	100*	96.10*
	
	Medicine	Fresh fruit	Rub the fruit juice against the body	Stop itching or insect bite	8.33	-

Kernels	Food	Open the pit using rocks as hammer and anvil	Snack food	Human nutrition	100	49.35

	Firewood	Collect dried wood	Fire wood	Home-use	67.86	87.01
	
Wood		Carving for agricultural tools	Hand tools, wheels of plough	Home-use	60.71	-
		
	Carving	Carving for cultural and home use purposes	Pestles, mortars, drums, stools, rosary, bowls, spoons, and canoes (large trees)	Home-use	94.05	54.55

Roots	Medicine			Swelling and gonococci healing	17.86	2.60

Only uses with FL greater than 5% in at least one of the study zone are displayed. Values with same superscript (*) are not significantly different (Fisher Exact Test; P < 5%)

Carvings of items such as spoons, plates and even shoes were reported to be used in the past but no longer in the dry Sudanian zone. The use of juice for making alcoholic beverages was no longer in practice in the Sudanian zone, whereas it was considered as potential use for *S. birrea *in the dry Sudanian zone.

#### Use value of Sclerocarya birrea

There was a significant difference in the overall use value and use values per category between study zones (Table 3). The highest overall use value was recorded in the dry Sudanian zone, which also displayed the greatest food, carving and medicinal use values. There was no significant gender-based difference in the overall use value of the species between the two zones. However, women use the species for firewood significantly more than men. In contrast, men cited uses for carving more often than women.

**Table 3:** Use value of *Sclerocarya birrea *per study zone in Benin

Use categories	Use values	Mann-Whitney		
	**Dry Sudanian zone (n = 84)**	**Sudanian zone (n = 77)**	**U**	**p-value**

Food	3.45 ± 0.12	1.95 ± 0.09	8870	< 0.001

Firewood	0.69 ± 0. 05	0.86 ± 0.03	6106	0.01

Carving	2.02 ± 0.15	0.53 ± 0.07	8633	< 0.001

Medicine	2.46 ± 0.13	1.65 ± 0.11	7835	< 0.001

Total	8.63 ± 0.28	5.00 ± 0.20	9037	< 0.001

The p values displayed indicate a significant difference in the overall and categorical use value of the species between study zones (P = 0.5)

Utilization of organs such as fruits, kernels and leaves were seasonal while the bark, the wood and roots could be used year-round. Although the cultural or worship value was not widespread, the wood of *S. birrea *was used frequently during funeral ceremonies in the dry Sudanian zone. *S. birrea *also plays an important role in animal rearing in both study zones. Leaves were often used as forage for livestock (sheep and goats) mainly at the beginning of rainy seasons when animals are penned to prevent the destruction of newly sowed crops.

## Discussion

### Uses and ethnoecological knowledge

S. *birrea *showed a multiple use pattern. Most of the recorded uses have been reported for the subspecies *caffra *(Marula) throughout its range [16]. However, the use of the species as a means for stimulating milk production in nursing women (galactagogue effect) appears to be a new finding in this study.

Some ethnic-specific names convey the way in which the species is perceived by local people and can be an indicator of how integrated the species is in their culture. The uses of *S. birrea *are currently only for subsistence purposes. The fruit and kernel have high commercial potential, though they are actually being underutilized. They are used almost only for a single subsistence purpose (Figure 4) while they are already commercially exploited in Southern Africa [16]. The low number of potential uses cited by informants indicates the limited awareness of the species' potential by local people. Our results show that some types of uses have become redundant (past uses), because the items formerly made by hand (spoons, plates, and rosaries) using the wood of *S.birrea *have been replaced by other items made from plastic or steel. This explains how specific knowledge about plant uses can be lost over time.

The majority of interviewees acknowledged that the *S. birrea *population has declined over time. The main reported causes by local people concur with others findings, where the decrease in rainfall and human pressure are well known causes of the decline in tree species in the sub or semi arid regions of West Africa [20]. Anthropogenic threats particularly land clearance for agricultural purposes stands as one of the main causes of the decline in both study zones. Moreover, these threats are enhanced in the semi-arid region by the use of the *S. birrea *for carving purposes.

### Use value difference within and between study zones

Our study revealed that the highest use values of the species were found in the dry Sudanian zone which also corresponds to where the species had the highest abundance in agroforestry systems. This suggests a positive relationship between plant abundance and use. These findings corroborate the "apparency hypothesis" which describes dominant, large and more abundant plant species as having the highest use values. This is not necessarily because of their inherent value, but because they are more visible or available to communities [6,7]. Populations in the Sudanian zone have alternative plant species which can meet their needs. *S. birrea *was replaced as a source of wood to be carved by another woody species more resistant and more suitable for some craft items (mortars, pestles) such as *Prosopis africana*. On the contrary, in the dry Sudanian zone, *S. birrea *is one of the most readily available, largest and most dominant trees found in agroforestry systems [14] and is used a lot in carving activities.

*S. birrea *was cited for use as firewood by women than by men. However, more men cited it's application for carving purposes (mainly agricultural tools) than women. This is consistent with the idea of that the division of labor by gender and gender specific use of landscape are likely to induce variation in the use pattern of a resource within a community [21]. This difference in knowledge was again apparent in the use of leaves as a stimulant for milk production in nursing women which was known almost exclusively by women.

One of the most widely reported factors likely to result in a difference in the use value of plant between communities is ethnicity [22,3,4]. However, the difference in use values, as we observed between zones, was not strongly related to the ethnic difference as in the case of African baobab in the same study zone [3]. For example the Gourmanché ethnic group present in both study zones did not display similarity in use value or in the method of use. Their use patterns were rather comparable to that of neighboring ethnic groups and were especially due to intercultural mixing which is a function of location.

### Importance of the species and implications for sustainable use

Despite the observed difference in the use patterns of *S. birrea*, all four of the important defined use categories were represented in both study zones. The medicinal use stood out as the most important use in both study zones. This form of use is consistent with previous findings [9,23] which concluded that extract of bark, root and leaves of Marula had a significant effect on bacterial growth. The reported treatment of diabetics with the bark reflects the local knowledge of the hypoglycemia inducing activity of the species as validated by scientific experiments [9,24].

The fruit and kernels of *S. birrea *appeared to be the most widely used organs (FL up to 100%), mainly by children and this confirmed that the species is an important component of the rural diet in several areas where it is found [25,26]. Furthermore, the nutrient analysis of the different components of the fruit showed that the juice of Marula contains 2 to 10 times the amount of ascorbic acid found in orange juice and a higher antioxidant capacity than other species commonly thought to be rich in antioxidant [27]. The kernels of *S. birrea *contain 47% to 56% fatty acid (from the dry weight) and contain many minerals that are beneficial for humans in relatively high proportions [28-30]. Its protein content (28 to 36.4%) is one of the highest found in more than 75 edible plants of the western Sahel [31,32,30].

In the dry Sudanian zone where the availability of large woody trees species is limited, *S. birrea *wood is of particular importance, as is evident by its multitudinous uses mainly in carving with higher FL compared to the Sudanian zone. This diversity of use of the wood has also been reported for Marula in southern and eastern Africa [33,16].

While demographic factors are most frequently reported as proximate causes of tropical deforestation [34], they are not independent of economic factors. If rates of biodiversity loss are to be slowed; economic incentives must play a central role in policy measures [35]. When a resource becomes economically important, the notion of property right can arise and may contribute to its conservation [36]. In the case of *S. birrea*, there is currently no economic return for local people as incentive to protect the species. Only immigrant carvers receive cash from the sale of some products (seats and mortars) that they carve from the wood. In spite of the local forestry protection law, they still consider the species as an open access resource and neglect to get the logging license required for harvesting the wood. This means that, they have unrestricted access to the species and it only profits them. In such situations, the "tragedy of the common" often prevails and overexploitation is a common result [37]. Thus, a framework for economic and effective exploitation of the species stands as one of the best policies by which to ensure its conservation.

## Conclusion

*S. birrea *subsp. *birrea *showed a multiple use pattern as do many of the important indigenous fruit tree species in West African rural areas. Its ability to provide people with two of their fundamental needs (food and medicine) and its potential to generate cash income make it a particularly important tree species which deserves more attention in terms of exploitation. Despite its current multitude of uses, *S. birrea *still remains underutilized in the light of the potential it has. For instance, the commercial use has been mentioned nowhere in the study zones although economic use of Marula in southern Africa has been strongly demonstrated in the literature. Currently, the quantity of fruits consumed is insignificant relative to the quantity of fruit produced each season (Gouwakinnou, unpublished data). All this unused fruit is doomed to rot, and a part of it is consumed by sheep, goats and wild animals. According to research conducted in South Africa, the value of Marula, as cash income through trade, varies from US$15 to US$166 per household per season of fruit collection and processing [38]. So far, it is still unclear how *S. birrea *subsp. *birrea *differ from *S. birrea *subsp. *caffra *and how this difference could be translated in their agronomic performance.

This study showed that while the use pattern of a species can be ethnically determined, many other factors such as the geographic location, the neighboring ethnic groups or the local availability (determined here by climatic conditions) of surrogate species in the proximate environment are also likely to influence the use value of a given plant species. Cautions should then be taken while drawing conclusions about the factors affecting the use pattern of a species and biodiversity in general, because environmental and context specific socio-cultural factors interact.

## Competing interests

The authors declare that they have no competing interests.

## Authors' contributions

GNG designed and performed the field work, analyzed and drafted the manuscript. AML and AEA gave conceptual advice, read and improved the drafted manuscript. BS supervised the work and improved the manuscript. All authors have read and approved the final manuscript.
